# Detection of *Vkorc1* single nucleotide polymorphisms indicates the presence of anticoagulant rodenticide resistance in Australia's introduced rats^†^

**DOI:** 10.1002/ps.8936

**Published:** 2025-05-30

**Authors:** Alicia F Gorbould, Quinton F Burnham, Michael T Lohr, Annette Koenders

**Affiliations:** ^1^ Conservation and Biodiversity Research Centre, School of Science, Edith Cowan University Joondalup Australia; ^2^ BirdLife Australia Melbourne Australia; ^3^ School of Medical and Health Sciences, Edith Cowan University Joondalup Australia

**Keywords:** anticoagulant, Australia, *Rattus*, resistance, rodenticide, *Vkorc1*

## Abstract

**BACKGROUND:**

Anticoagulant rodenticides (ARs) are used globally to manage pest rodent populations. However, resistance to ARs in target rodent populations challenges pest control efforts and can increase risks to nontarget species. Resistance is frequently associated with nonsynonymous single nucleotide polymorphisms (nsSNPs) in the *Vkorc1* gene, and this study carried out the first *Vkorc1* survey of introduced rats on the Australian mainland.

**RESULTS:**

We identified three species of introduced rat using the cytochrome *b* gene across Brisbane, Melbourne, Perth and Sydney: *Rattus rattus* (Linnaeus 1758) (Lineage I); *Rattus norvegicus* (Berkenhout 1769); and *Rattus tanezumi* (Temminck 1844) (Lineage II). Three nsSNPs were detected in the *Vkorc1* gene: *Tyr25Phe*, *Trp59Arg* and *Phe55Ile*. The mutation *Tyr25Phe*, which is associated with resistance to ARs, was identified in 58 of 108 *R. rattus* (53.7%) and one of 31 *R. tanezumi* (3.2%). It has been suggested that the mutation *Trp59Arg* (detected in two *R. rattus*) can increase susceptibility to haemorrhage, whereas the mutation *Phe55Ile* (identified in only one *R. rattus*) has not been reported previously. No nsSNPs were identified in *R. norvegicus*.

**CONCLUSION:**

This is the first update to the resistance status of introduced rats on the Australian mainland since the 1970s and the first to employ genetic screening. The widespread occurrence of *Tyr25Phe* in urbanized areas of Australia suggests potential resistance to ARs is common in *R. rattus*. However, practical resistance conferred by *Tyr25Phe* needs further investigation as does the role of hybridization in the transfer of resistance from the *R. rattus* to the *R. tanezumi* nuclear genome. © 2025 The Author(s). *Pest Management Science* published by John Wiley & Sons Ltd on behalf of Society of Chemical Industry.

## Graphical abstract

Introduced rat species in Australia were tested for *Vkorc1* gene mutations associated with anticoagulant rodenticide resistance. The widespread occurrence of *Tyr25Phe* suggests potential resistance is common in *Rattus rattus*. Image created with BioRender.com

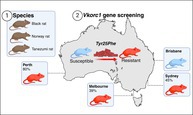

## 1 INTRODUCTION

Anticoagulant rodenticides (ARs) have been the mainstay of rodent pest control since their advent in the 1940s.^1^, ^2^ ARs target the liver enzyme vitamin K epoxide reductase (VKOR), which plays an important role in the vitamin K cycle that is essential for normal blood clotting function;^3^, ^4^, ^5^ the disruption of which can result in death by internal haemorrhage.^6^ ARs have been used successfully to control rodents, yet the unchecked use of ARs can select for a gene mutation which confers resistance.^7^ Nonsynonymous single nucleotide polymorphisms (nsSNPs) in the *vitamin K epoxide reductase complex subunit 1* (*Vkorc1*) gene^8^ are responsible for the change of native amino acids in VKOR that is associated with resistance in rodents. These mutations, combined with other factors contributing to eradication failure, can hinder rodent control by reducing the effectiveness of ARs in managing pest rodent populations.^7^ Resistance to first‐generation ARs (FGARs) was identified as early as 1958^9^ and has been reported widely in *Rattus rattus* (Linnaeus 1758),^10^, ^11^, ^12^, ^13^ *Rattus norvegicus* (Berkenhout 1769),^9^, ^10^, ^14^, ^15^, ^16^ *Rattus tanezumi* (Temminck 1844)^17^ and *Mus musculus* (Linnaeus 1758).^10^, ^12^, ^18^ In response to the spread of resistance, second‐generation ARs (SGARs) – which are more potent than FGARs – were developed,^1^ yet now there is also evidence of resistance to SGARs in pest rodent populations.^18^, ^19^, ^20^, ^21^, ^22^, ^23^, ^24^, ^25^, ^26^

Poisoning by ARs of companion animals^27^, ^28^, ^29^ and children under five^30^, ^31^ have been reported and is concerning, yet perhaps an even greater issue is the contamination of entire food chains with SGARs owing to their high potential for bioaccumulation and biomagnification.^32^, ^33^ The persistent nature of SGARs means that predators can be exposed not only through the consumption of moribund or dead rodents,^1^, ^34^, ^35^ but also through the consumption of prey which has been secondarily exposed to ARs,^32^ or exposed through environmental contamination of soil^1^, ^32^ or waterbodies.^32^

The Australian mainland provides a unique opportunity to study AR resistance: it is a large island and FGARS and SGARS are freely available to the Australian public. Australia's expansive size and geographical isolation present a partial barrier to rodent gene flow into and out of the country and between Australian cities. Across Europe,^36^, ^37^ North America^36^ and Britain,^38^ *R. norvegicus* (Norway or brown rat) is now the dominant pest rat and resistance to ARs is common in this species.^39^ Despite both *R. norvegicus* and *R. rattus* being introduced to mainland Australia with European exploration,^40^, ^41^, ^42^ *R. rattus* rather than *R. norvegicus* is the predominant species and resistance in this species is less extensively researched.^43^ Additionally, with SGARs widely available to the Australian public^44^ with few restrictions on use,^34^, ^44^ lethal doses of SGARs have been detected in native Australian birds that routinely feed on rodents, such as the tawny frogmouth (*Podargus strigoides* Latham 1801),^34^ Australian boobook (*Ninox boobook*, Latham 1801)^34^, ^45^ and eastern barn owl (*Tyto javanica* Gmelin 1788).^34^ Biomagnification of SGARs through the food chain has meant that it is also found in apex predators that are not rodent‐centric predators, such as the Tasmanian wedge‐tailed eagle (*Aquila audax fleayi* Condon & Amadon 1954)^46^ and powerful owl (*Ninox strenua* Gould 1838),^47^ and a broad range of nonpredatory and/or non‐avian taxa including frogs and toads,^48^ reptiles^49^ and possums.^50^

Despite the long‐term use of ARs in Australia and the accumulation of ARs in nontarget species, the resistance status of introduced rats and the efficacy of ARs in controlling rat populations in Australia was unknown before this study. In the 1970s, a feeding trial involving *R. rattus* and *R. norvegicus* captured from the Sydney region established *R. norvegicus* as susceptible and *R. rattus* as resistant to the first‐generation AR, warfarin.^13^ The only genetic investigation into resistance in Australian rodents was for house mouse (*M. musculus*) from Browse Island and Perth, Western Australia,^51^ which found no evidence of resistance. There have been no published genetic investigations into AR resistance of rats on the Australian mainland and their resistance status has not been tested since the first feeding trial.

By screening the gene that encodes for VKOR in rats from the four largest urban centres of the Australian mainland (Brisbane, Melbourne, Perth and Sydney) for any mutations that confer resistance, this study aims to provide an update on resistance status and provide a baseline for future monitoring of resistance in Australia's rat populations. Given Australia's relative isolation from other populated areas in the world, the geographical separation between its major cities, the relatively recent introduction of rats to the continent, and extensive and uncontrolled use of ARs, this information may provide some insight into how AR resistance arises and spreads. This information can be used to minimize exposure of nontarget biota to ARs and to improve the efficacy of rodent control programmes both in Australia and internationally.

## 2 MATERIALS AND METHODS

### 2.1 Rat tissue sampling

Rat tail samples (*n =* 169) were collected between 2021 and 2024 by pest control officers (PCOs) as part of the regular operation of a private pest control business. Rats were captured from Brisbane, Melbourne, Perth and Sydney using physical trapping methods. Tails were removed from the deceased animal at the base and stored in absolute ethanol at 4° C before DNA extraction. Some samples collected in Perth were stored at −20° C (not fixed in ethanol) including an additional 23 whole animals trapped and donated by members of the public between 2018 and 2021.

### 2.2 Tissue preparation and DNA extraction

Muscle tissue was removed from each tail, avoiding the skin to minimize contamination originating at the point of collection, and to limit collagen content which impedes DNA yield.^52^ For tissue stored in ethanol, excised muscle portions were maintained on a rocking platform at ambient temperature for 30 min in ultrapure water (Sartorius AG, Göttingen, Germany) to displace ethanol residue and to rehydrate samples. This was repeated another two times with a water change each time to prepare samples for DNA extraction. DNA was extracted using the DNeasy Blood and Tissue kit (Qiagen, Hilden, Germany).

### 2.3 Polymerase chain reaction (PCR) amplification

Polymerase chain reaction (PCR) was used to amplify the cytochrome *b* gene and the three coding regions of the *Vkorc1* gene (exons 1, 2 and 3). Cytochrome *b* amplification was carried out to determine species identity using universal primers L14723 and H15915^53^, ^54^ under the following cycling conditions: initial activation at 94 °C for 2 min; 45 cycles of 94 °C for 30 s, a touchdown of 60–46.8 °C (0.3 °C per cycle) for 30 s, extension of 72 °C for 1 min 15 s and a final elongation at 72 °C for 10 min. For the *Vkorc1* gene, two primer sets described previously^55^ were used to amplify the three coding regions of the gene: one primer set was used to amplify exon 1 and 2 together (exon1‐forward and exon2‐reverse), and a second set was used to amplify exon 3 (exon3‐forward and exon3‐reverse). Where amplification of the *Vkorc1* gene using two primer sets was not successful, an additional set of primers was used to amplify each of the exons individually as specified by Grandemange *et al*.^55^ Exons 1 and 2 were amplified under the following PCR cycling conditions: 94 °C for 2 min; 45 cycles of 94 °C for 30 s, a touchdown from 63 to 58.6 °C (0.1 °C per cycle) for 30 s and 72 °C for 1 min 15 s, with a final extension at 72 °C for 10 min. Exon 3 was amplified under the following conditions: 94 °C for 2 min; 45 cycles of 94 °C for 30 s, 63 °C for 30 s and 72 °C for 1 min, with a final extension of 72 °C for 10 min.

For both cytochrome *b* and *Vkorc1* amplification, each PCR mixture contained: 12.5 μL Platinum™ Green Hot Start PCR Master Mix (2X) (Thermo Fisher Scientific, Waltham, MA, USA); 0.5 μL each of the forward and reverse primers (10 μm); 0.5–2 μL genomic DNA; and nuclease‐free water up to a volume of 25 μL. Gel electrophoresis confirmed successful amplification of PCR products on a 1.5% agarose gel stained with SYBR™ Safe DNA Gel Stain (Invitrogen/ Thermo Fisher Scientific).

### 2.4 DNA sequencing, editing and analysis

Successfully amplified products were sequenced in both forward and reverse directions by the Australian Genome Research Facility (AGRF). Sequences were edited in geneious prime (2023.2.1).

Species identity was confirmed using the Nucleotide Basic Local Alignment Search Tool (blastn), requiring ≥98% sequence identity for the cytochrome *b* gene. For *R. rattus* or *R. tanezumi* identified individuals, sequences were aligned to the *Vkorc1* gene on chromosome two of the NCBI reference sequence for *R. rattus* (NC_046155). No NCBI reference sequence for *R. tanezumi* exists at the time of this study for the *Vkorc1* gene, and the same sequence was used for both species owing to the cryptic nature of the ‘*Rattus rattus* species complex’ and the relatively short divergence time between *R. rattus* and *R. tanezumi* (≈300 000 years).^56^ For individuals identified as *R. norvegicus*, sequences were aligned to the ENSEMBL reference sequence for the *Vkorc1* gene (ENSRNOG00000050828; NCBI Gene ID 309004) as used previously by Yiğit *et al*.^57^ Sequences in this study were compared to their respective genomic references to identify SNPs and any corresponding amino acid changes.

## 3 RESULTS

### 3.1 Species identification

Molecular verification of species, as delineated by the cytochrome *b* gene, indicated that species identification undertaken by PCOs was not always reliable (data not presented). *R. rattus* was the initial target species because it is the most common and widespread species on the Australian mainland; however, the scope of this study was broadened to include any introduced rat species as PCOs had difficulty distinguishing *R. rattus* from *R. norvegicus*.

**Figure 1:**
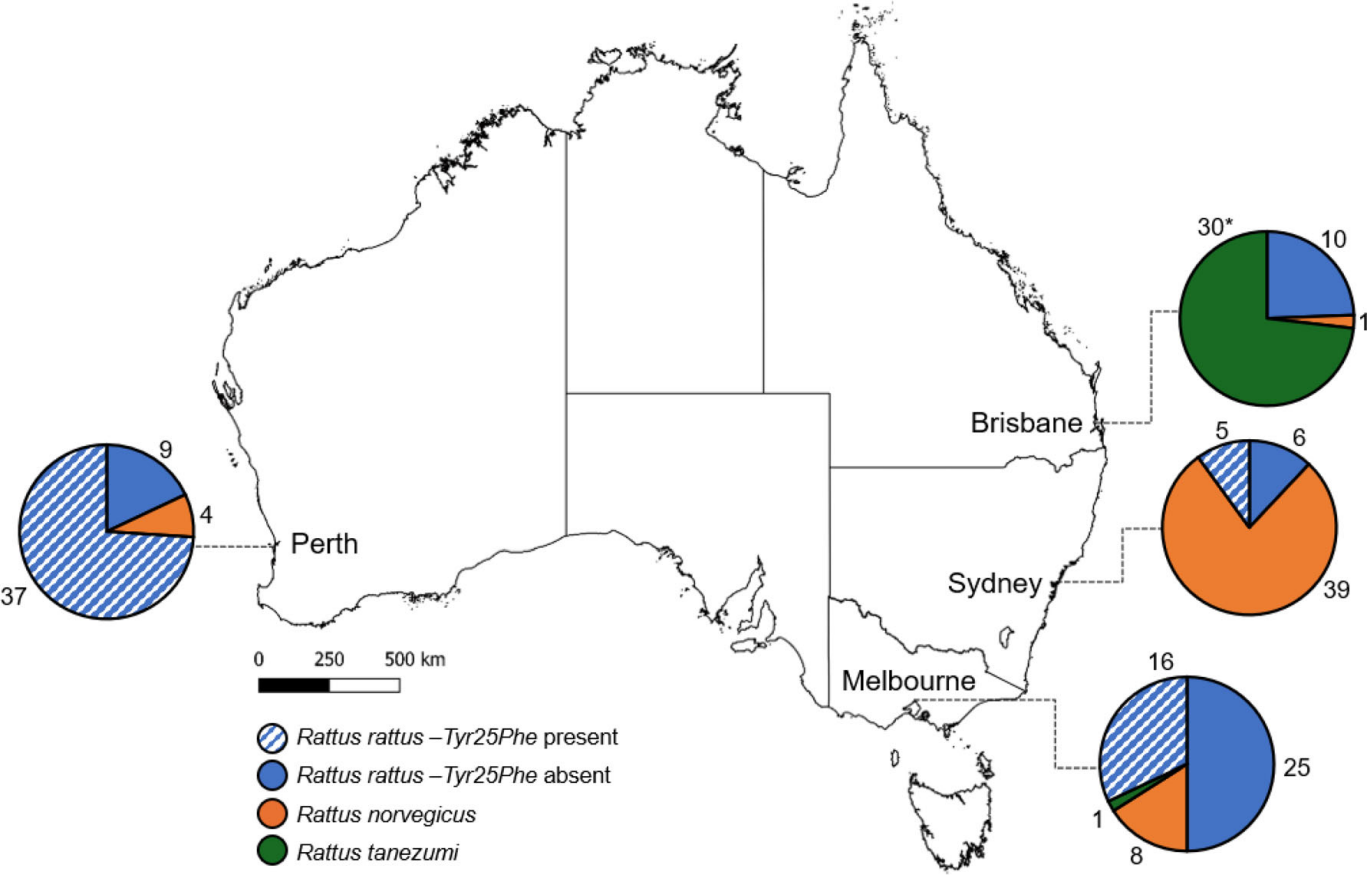
Species identity of samples collected from the Australian cities Brisbane, Melbourne, Perth and Sydney (total *n =* 192), identified using the cytochrome *b* gene. The occurrence of the *Vkorc1* mutation associated with anticoagulant resistance (*Tyr25Phe*) in *Rattus rattus* is indicated with the hatched pattern. The asterisk indicates the detection of one *Rattus tanezumi* in Brisbane carrying the *Tyr25Phe* mutation.

Of the 192 specimens sequenced for the cytochrome *b* gene, the predominant species was *R. rattus* (109; 56.8%), followed by *R. norvegicus* (52; 27.1%) and *R. tanezumi* (31; 16.1%) (Fig. 1). The proportion of each species collected varied across the capital cities (Fig. 1).

### 3.2 *Vkorc1* sequencing

Of the 192 specimens sequenced for the cytochrome *b* gene, 191 specimens were successfully sequenced for the coding regions of the *Vkorc1* gene. Coverage with both forward and reverse sequences for each exon was not always successful, in which case a single direction sequence was used.

A total of nine *Vkorc1* polymorphisms were recorded across the three rat species: three nsSNPs and six synonymous SNPs (sSNPs) (Table 1). All nsSNPs were identified in *R. rattus*, one of which was also found in *R. tanezumi* (Table 1). No double mutants were recorded and no nsSNPs were recorded in *R. norvegicus*.

**Table 1:** Single nucleotide polymorphisms (SNPs) of the *Vkorc1* gene in *Rattus* specimens from the Australian mainland and their associated amino acid changes compared to the wild‐type (*Rattus rattus* and *Rattus tanezumi* NCBI reference: NC_046155; *Rattus norvegicus* EMSEMBL reference: ENSRNOG00000050828). Values indicate SNP occurrence as a percentage of total sample size for each species, rounded up to one decimal place. Rows in bold indicate nonsynonymous SNPs

						*Rattus rattus*			*Rattus norvegicus*			*Rattus tanezumi*		
						*n =* 108			*n = 52*			*n = 31*		
Mutation	Exon	Nt Position	Codon Position	Codon WT	Codon Mutation	Heterozygous %	Homozygous %	Total %	Heterozygous %	Homozygous %	Total %	Heterozygous %	Homozygous %	Total %
*Arg12Arg*	1	36	12	CCG	TCG	0.0	0.0	0.0	1.9	0.0	1.9	0.0	0.0	0.0
***Tyr25Phe***	**1**	**74**	**25**	**TAC**	**TTC**	**17.6**	**36.1**	**53.7**	**0.0**	**0.0**	**0.0**	**3.2**	**0.0**	**3.2**
*Ala41Ala*	1	123	41	GCA	GCG	12.0	79.6	91.6	0.0	0.0	0.0	3.2	93.5	96.7
***Phe55Ile***	**1**	**163**	**55**	**TTC**	**ATC**	**0.9**	**0.0**	**0.9**	**0.0**	**0.0**	**0.0**	**0.0**	**0.0**	**0.0**
***Trp59Arg***	**2**	**175**	**59**	**TGG**	**AGG**	**0.0**	**1.9**	**1.9**	**0.0**	**0.0**	**0.0**	**0.0**	**0.0**	**0.0**
*His68His*	**2**	**204**	68	GTG	ATG	0.0	0.0	0.0	0.0	100.0	100.0	0.0	0.0	0.0
*Ile82Ile*	**2**	**246**	82	TAT	AAT	0.0	0.0	0.0	26.9	1.9	28.8	0.0	0.0	0.0
*Leu94Leu*	2	280	94	CTA	TTA	11.1	4.6	15.7	0.0	0.0	0.0	32.3	12.9	45.2
*Ala143Ala*	3	429	143	GCA	GCG	15.7	1.9	17.6	0.0	0.0	0.0	32.3	9.7	42.0

*Note*: The colours in the table match the corresponding species colours shown in (Fig. 1).

#### 3.2.1 Rattus rattus

Of the 108 *R. rattus* individuals screened for the *Vkorc1* gene, >90% of carried at least one mutation (either nsSNPs or sSNPs) (Table 1). A total of three nsSNPs were identified in *R. rattus*: *Tyr25Phe* and *Phe55Ile* in the exon 1 coding region, and *Trp59Arg* in the coding region of exon 2. A total of 58 individuals (19 heterozygotes, 39 homozygotes) (53.7%) were included in the count of resistant individuals found with the *Tyr25Phe* mutation. Geographical distribution of this mutation varied between cities (Fig. 1) with Perth showing the highest frequency (37 of 46; 80.4%), followed by Sydney (five of 11; 45.5%) and Melbourne (16 of 41; 39.0%), and no detections were recorded in Brisbane (zero of 10). *Phe55Ile* was detected in one individual from Sydney (heterozygous) and *Trp59Arg* was detected in two individuals from Melbourne (both homozygotes).

Three sSNPs were detected in *R. rattus*: *Ala41Ala* in the coding region of exon 1, *Leu94Leu* in the coding region of exon 2, and *Ala143Ala* in the coding region of exon 3. *Ala41Ala* was the most common in Perth (45 of 46; 97.8%), Melbourne (39 of 41; 95.1%) and Sydney (10 of 11; 90.9%), followed by Brisbane (five of 10; 50.0%). *Leu94Leu* and *Ala143Ala* were less frequently detected in all cities except Perth, where neither was detected. *Leu94Leu* was the most frequent in Sydney (seven of 11; 63.6%), followed by Brisbane (two of 10; 20.0%) and Melbourne (eight of 41; 19.5%). *Ala143Ala* was detected the most frequently in Sydney (seven of 11; 63.6%), followed by Brisbane (three of 10; 30.0%) and Melbourne (nine of 41; 22.0%).

#### 3.2.2 Rattus norvegicus

No nsSNPs were identified in this species, yet a total of three sSNPs were identified in *R. norvegicus*, none of which were detected in the other sampled species: *Arg12Arg* in the exon 1 coding region, *His68His* in the exon 2 coding region, and *Ile82Ile* in the coding region of exon 3 (Table 1). *Arg12Arg* was identified in a single specimen from Sydney (one of 39; 2.6%) (heterozygous). *His68His* was present in all *R. norvegicus* from all cities (all homozygotes) whilst *Ile82Ile* was present in Melbourne (four of eight; 50.0%) (all heterozygotes) and Sydney (11 of 39; 28.2%) (one homozygote, 10 heterozygotes), but not in Brisbane or Perth.

#### 3.2.3 Rattus tanezumi

*Rattus tanezumi* (Lineage II) were collected from Brisbane (*n* = 30) and Melbourne (*n* = 1) and a total of four mutations in the coding regions of the *Vkorc1* gene were recorded in these specimens: a single nsSNP (*Tyr25Phe*; 1 of 31), and three sSNPs (Table 1) (*Ala41Ala*, *Leu94Leu* and *Ala143Ala*). The specimen carrying the *Tyr25Phe* mutation (heterozygous) was collected in Brisbane. Of the 31 *R. tanezumi* specimens in this study, 30 possessed *Ala41Ala* including the specimen from Melbourne.

## 4 DISCUSSION

Anticoagulant rodenticides have been used in Australia for ≥50 years and continue to be used with minimal monitoring,^46^ despite some difficulty in controlling rats with rodenticides first being reported in the early 1970s.^13^ Recently concerns have been raised regarding the impact that bioaccumulation and biomagnification of ARs are having on Australian wildlife;^34^, ^45^, ^46^, ^48^, ^58^ mirroring observations from Europe and other regions of the world.^32^, ^35^, ^59^, ^60^, ^61^, ^62^ The present work is the first to identify genetic evidence for AR resistance in Australia's introduced rats. As the rats in Australia are relatively recent introductions and have little opportunity for genetic transfer across broad geographical regions, they could provide an interesting case study of how often the mutations may arise, the conditions that may promote and facilitate their spread, and how the situation can best be managed.

In SNP screening of the *Vkorc1* gene, introduced rats in four major capital cities of Australia were found to carry the nonsynonymous mutations *Tyr25Phe*, *Phe55Ile* and *Trp59Arg*. The *Tyr25Phe* variant is associated with the resistant phenotype^20^, ^43^ and was identified in over a quarter of all rats (30%) and was predominant in *R. rattus* (53.7%). In *R. rattus*, this variant was the most frequent in Perth (80.4%), followed by Melbourne (39.0%) and Sydney (45.5%), and was not detected in any *R. rattus* collected from Brisbane but was detected in a single *R. tanezumi* from this location. *R. rattus* also was found to carry the nonsynonymous mutations *Phe55Ile* and *Trp59Arg* which have not undergone testing to establish whether they are associated with resistance in rats. It is possible that *Phe55Ile*, detected in one *R. rattus* specimen from Sydney, confers resistance to ARs because it has been proposed as a key warfarin‐binding residue in VKOR and its effect varies from complete resistance to complete susceptibility to warfarin in humans.^63^ However, this mutation has not been reported previously in rodents and the effect of this mutation in *R. rattus* is unclear and warrants further investigation. However, *Trp59Arg*, detected in two *R. rattus* specimens from Melbourne, is associated with decreased VKOR activity^43^ and may also be associated with increased haemorrhage, even without exposure to ARs.^8^ *Trp59Arg* has been reported previously in *R. norvegicus* in Argentina,^8^ in *R. rattus* in Spain and France,^43^ and more recently in *R. rattus* in Italy^64^ and in mice in the Czech Republic.^65^

Unlike in Europe, where resistance is commonly encountered in *R. norvegicus*, we did not detect any mutations which may confer resistance in this species in Australia and all mutations that we did detect have been reported previously.^57^ This situation mirrors that of New Zealand, where *R. norvegicus* exhibited no nonsynonymous mutations.^66^ New Zealand shares a similar introductory history and pattern of AR use, and the absence of nonsynonymous mutations may be a result of inadequate *Vkorc1* variation in the first founding individuals or the removal of warfarin‐resistant *R. norvegicus* with the advent of SGARs, as proposed by Cowan *et al*.^66^ Additionally, *R. rattus* has both a wider distribution and broader diet than *R. norvegicus*,^67^ and frequent consumption of plant matter^68^ may permit the maintenance of *Vkorc1*‐mediated resistance through adequate dietary vitamin K_1_ intake, because *Tyr25Phe* is associated with reduced VKOR activity.^43^ *Vkorc1*‐mediated resistance may be functionally difficult to maintain for *R. norvegicus* in Australia because the preferred habitat of *R. norvegicus* (sewers, ground level of buildings,^41^ landfill sites^69^) may prevent increased physiological demands for vitamin K_1_ from being met through the diet.

The implications of the *Tyr25Phe* variant for rodent management are speculative, as resistance has only been tested *in vitro*. This mutation was first identified on a farm in Spain where control of *R. rattus* homozygous for *Tyr25Phe* had failed, despite intensive treatment with bromadiolone.^20^ By contrast, Damin‐Pernik *et al*.^43^ suggest that effective resistance of this mutation is low, and practical resistance will only be to warfarin. If this mutation only conferred resistance to warfarin, we would expect the frequent use of SGARs – which are more commonly available to the Australian public than FGARs – to diminish *Tyr25Phe* to levels lower than identified here; levels that we detected were lower than detected previously in *R. rattus* in Spain,^20^ yet higher than those detected on average in the USA,^70^ and considerably higher than in *R. rattus* in New Zealand.^66^ It is unclear whether less potent SGARs, difenacoum and bromadiolone, would be effective against this mutation and the resistance of the *Tyr25Phe* variant to other FGARs available in Australia (diphacinone and coumatetralyl) needs evaluation. However, where resistant alleles have been detected in Australia, the continued use of first‐generation rodenticides, particularly warfarin, will promote *Tyr25Phe* in *R. rattus* populations. Where *Vkorc1*‐mediated resistance was low (Brisbane), the use of SGARs appears to be superfluous. As evidence for *Vkorc1*‐mediated resistance was not found in *R. norvegicus*, FGARs can, and arguably should, be used to limit the likelihood of nontarget poisonings when targeting this species. The use of more potent SGARs, such as brodifacoum, difethialone and flocoumafen, is likely to be unnecessary in the control of introduced rats and their regular use in Australia continues to be at the expense of the environment. The resistance status of other species, such as mice, should also be carefully considered especially in control efforts that target multiple species. The choice of ARs could inadvertently promote resistance in one species or another and highlights the complexity of making informed decisions when using anticoagulant rodenticides.

The sympatric occurrence of *R. tanezumi* and *R. rattus* on the Australian mainland presents an opportunity for hybridization, and heritable acquisition of *Vkorc1‐*mediated resistance from *R. rattus* to *R. tanezumi*. Introgression may be the reason that the *Tyr25Phe* mutation was identified in one *R. tanezumi* individual, and why all synonymous *Vkorc1* mutations in *R. tanezumi* were likewise observed in *R. rattus*. Mating of the two lineages generally results in the swamping out of the *R. tanezumi* nuclear genome (particularly where *R. rattus* mates are more commonly encountered than *R. tanezumi* ^71^) to the extent that both species are eventually indistinguishable in their nuclear genome.^72^ However, the mtDNA identity of *R. tanezumi* can be retained through this process^71^ and it has also been proposed that the opposite can occur.^73^ It is important to understand the potentially convoluted genetic histories of hybridized pest rats, which may influence resistance to ARs^72^ or pose an increased risk for the harbour and spread of zoonotic disease.^72^ The use of nuclear microsatellite and nuclear gene sequencing, as carried out by Conroy *et al*.,^72^ Lack *et al*.^71^ and Thomson *et al*.^73^ would allow for an understanding of the extent of hybridization between these species and is recommended for future investigations of *Rattus* spp. in Australia.

Further geographical evaluation of *Vkorc1*‐mediated resistance in rats and mice, to complement the work already conducted in mice in Perth,^51^ would help to complete the picture for rodents and their management on the Australian mainland, particularly because rodents are currently controlled in an indiscriminate way. The broad‐scale application of ARs, and the lack of discernment between species, shows that management approaches in Australia are not tailored; this situation is not unique to Australia and similar issues have been reported in Europe^43^, ^74^ and New Zealand.^75^ This emphasizes the importance of assessing the resistance status of both rats and mice and the careful selection of an appropriate AR (or an alternative).

Other types of resistance contribute to practical issues in controlling rodents such as innate differences in resistance between sex and species, avoidance behaviours, microbiome‐derived resistance, and metabolic resistance mediated by cytochrome P450 genes.^76^ It is feasible that prolonged exposure of Australia's introduced rats to ARs has led to a combination of these types of resistance, including in *R. norvegicus* for which we found no nonsynonymous *Vkorc1* mutations. The full extent of resistance should be further investigated with field trials in addition to the on‐going monitoring of *Vkorc1* mutations in *Rattus* spp.; monitoring is an important component of a long‐term pest management programme given low‐grade resistance can lead to resistance to second‐generation ARs.^26^

## 5 CONCLUSION

This is the first survey of *Vkorc1*‐mediated resistance in introduced rats on the Australian mainland and the first update to AR resistance status in introduced rats since the 1970s. The prevalence of the *Tyr25Phe* mutation, which previously has been associated with AR resistance, suggests the occurrence of AR resistance in *R. rattus*, but not in *R. norvegicus*. In the absence of field trials, it is unclear what practical levels of resistance may be encountered, and it is possible that other types of resistance (such as metabolic resistance) underlie a resistant phenotype. However, treatment of populations with warfarin is likely to promote this mutation in *R. rattus* populations, and the use of second‐generation ARs to control *R. norvegicus* continues to pose an unnecessary risk to nontarget species. A third introduced species collected on the mainland, *R. tanezumi*, may be part of a collective gene pool with *R. rattus*, and the role of hybridization in the transmission of resistance remains to be investigated further.

## Data Availability

The data that support the findings of this study are available from the corresponding author upon reasonable request.
